# Prediction of the 10-year risk of atherosclerotic cardiovascular disease in the Korean population

**DOI:** 10.4178/epih.e2023052

**Published:** 2023-05-12

**Authors:** Sangwoo Park, Yong-Giun Kim, Soe Hee Ann, Young-Rak Cho, Shin-Jae Kim, Seungbong Han, Gyung-Min Park

**Affiliations:** 1 Department of Cardiology, Ulsan University Hospital, University of Ulsan College of Medicine, Ulsan, Korea; 2 Department of Cardiology, Dong-A University Hospital, Busan, Korea; 3 Department of Biostatistics, Korea University College of Medicine, Seoul, Korea

**Keywords:** Cardiovascular disease, Risk assessment, Risk factors, Primary prevention

## Abstract

**OBJECTIVES:**

Proper risk assessment is important for the primary prevention of atherosclerotic cardiovascular disease (ASCVD). However, no validated risk prediction tools are currently in use in Korea. This study sought to develop a 10-year risk prediction model for incident ASCVD.

**METHODS:**

Using the National Sample Cohort of Korea, 325,934 subjects aged 20-80 years without previous ASCVD were enrolled. ASCVD was defined as a composite of cardiovascular death, myocardial infarction, and stroke. The Korean atherosclerotic cardiovas cular disease risk prediction (K-CVD) model was developed separately for men and women using the development dataset and validated in the validation dataset. Furthermore, the model performance was compared with the Framingham risk score (FRS) and pooled cohort equation (PCE).

**RESULTS:**

Over 10 years of follow-up, 4,367 ASCVD events occurred in the overall population. The predictors of ASCVD included in the model were age, smoking status, diabetes, systolic blood pressure, lipid profiles, urine protein, and lipid-lowering and blood pressure-lowering treatment. The K-CVD model had good discrimination and strong calibration in the validation dataset (time-dependent area under the curve=0.846; 95% confidence interval, 0.828 to 0.864; calibration χ^2^=4.73, goodness-of-fit p=0.32). Compared with our model, both FRS and PCE showed worse calibration, overestimating ASCVD risk in the Korean population.

**CONCLUSIONS:**

Through a nationwide cohort, we developed a model for 10-year ASCVD risk prediction in a contemporary Korean population. The K-CVD model showed excellent discrimination and calibration in Koreans. This population-based risk prediction tool would help to appropriately identify high-risk individuals and provide preventive interventions in the Korean population.

## GRAPHICAL ABSTRACT

[Fig f3-epih-45-e2023052]

## INTRODUCTION

Atherosclerotic cardiovascular disease (ASCVD) is the leading cause of premature death worldwide and has become an important burden on the healthcare system in recent decades [1]. Regarding decision-making for the primary prevention of ASCVD, proper global risk prediction is important for identifying high-risk individuals who are more likely to benefit from preventive interventions [2].

Currently, many guidelines on the prevention of ASCVD strongly recommend the use of risk assessment tools for risk stratification [3,4]. However, the most widely used tools—namely, the Framingham risk score (FRS), pooled cohort equation (PCE), and Systemic Coronary Risk Estimation (SCORE)—were mainly developed based on Western populations [5-7]. However, given the ethnic and geographic differences in risk factor profiles and the incidence of ASCVD between Western and non-Western countries, directly applying these models to populations other than those from which the tools were originally developed may lead to the overestimation or underestimation of ASCVD risk [4].

Korea is among the countries with the lowest cardiovascular mortality rates and the fastest decline in age-adjusted cardiovascular mortality worldwide [8]. In this regard, applying risk assessment tools developed based on Western populations could overestimate ASCVD risk and the potential benefits of preventive treatment in the Korean population [9,10]. However, there is still no well-validated model based on data from long-term cohort studies in the Korean population. Therefore, no risk prediction tools are currently endorsed by Korean guidelines to estimate the 10-year risk of ASCVD; instead, therapeutic goals set based on the total number of risk factors are still used to guide decision-making for medical therapy, which may lead to overly simplified conclusions [11,12].

To address these issues, we aimed to develop and validate a risk prediction model to estimate the 10-year ASCVD risk in individuals without previous ASCVD using a representative nationwide cohort of the Korean population.

## MATERIALS AND METHODS

### Data sources

This study was based on the National Health Insurance Service–National Sample Cohort (NHIS-NSC), which is a populationbased cohort established by the National Health Insurance Service (NHIS) in Korea [13]. The most recent version of the NHIS-NSC was constructed by systematic stratified random sampling with proportional allocation to ensure the representativeness of the cohort. The NHIS-NSC, comprising 2.1% of the total eligible Korean population in 2006, was followed for 13 years until 2019 unless participants became ineligible due to death or emigration. The cohort contains databases with information on participants’ insurance eligibility (including demographic data, socioeconomic variables, and causes of death based on death certificates from the National Statistical Office), healthcare claims data (including diagnosis, treatment, procedure, surgery, and prescribed medication), and general health examinations. The NHIS is a compulsory social insurance system that covers the entire Korean population. In addition to the single-payer insurance system, a fee-for-service payment system that has been the standard reimbursement model for outpatient care and the majority of inpatient care enables the collection of comprehensive data regarding healthcare usage at the national level. The general health examination database comprises information on nationwide health examinations conducted by the NHIS [13]. All insured Korean adults aged 40 years or older are eligible for the general health screening program, which is conducted biennially (annually for manual workers). An insured employee or a householder of the insured self-employed can receive a general health examination regardless of their age [14]. In 2009, major changes were made to the content of the general health examinations, and since then, high-density lipoprotein cholesterol (HDL-C) and triglyceride levels have been included in the laboratory test items in addition to total cholesterol. The inclusion of lipid profiles in the general health examination enabled the potential use of the NHIS-NCS to predict ASCVD in the Korean population.

### Study population

**Figure 1.**
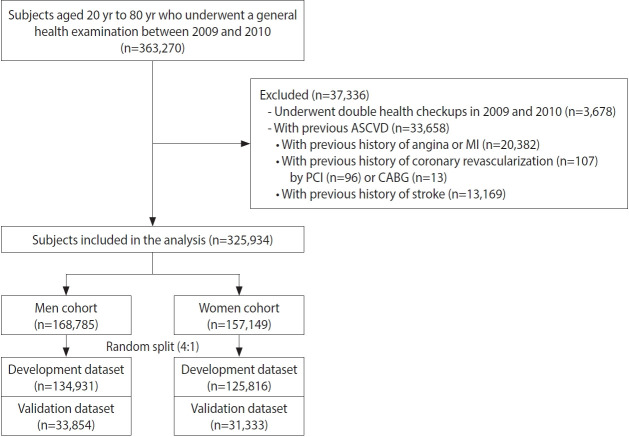
Study flowchart. A flowchart showing the subject selection process, presenting the numbers of subjects excluded from and enrolled in the current study. ASCVD, atherosclerotic cardiovascular disease; CABG, coronary artery bypass grafting; MI, myocardial infarction; PCI, percutaneous coronary intervention.

To develop a 10-year ASCVD risk prediction model in a representative Korean population, we identified 363,270 subjects aged 20 years to 80 years who underwent a general health examination between January 2009 and December 2010 from the NHIS-NSC database. This time period potentially provided 10 years of median follow-up until the end of 2019. For the 3,678 subjects who underwent general health examinations in both years, the records of health checkups from 2009 were included and the records from 2010 were excluded. Subjects were further excluded if their claims records indicated that they had a history of ASCVD before the index health checkup. Accordingly, 33,658 subjects were sequentially excluded based on the following criteria: (1) previous history of angina or myocardial infarction (MI; n = 20,382); (2) previous history of coronary revascularization (n= 107; percutaneous coronary intervention [n = 96] or coronary artery bypass grafting [n= 13]); and (3) previous history of stroke (n= 13,169). Subjects diagnosed with atrial fibrillation or those taking antiplatelet drugs for reasons other than ASCVD were not excluded from the study population. Finally, 325,934 subjects were included in this study (Figure 1).

### Study variables

All relevant variables associated with ASCVD risk were included in this study, as shown in Table 1. Most variables, such as anthropometric measurements (body mass index, waist circumference, and blood pressure [BP]), questionnaires regarding past medical history, family history, and health behavior (smoking and physical activity), and laboratory test results were obtained from the general health examination results. Comorbidities such as hypertension, diabetes, and hyperlipidemia were identified using the International Classification of Diseases, 10th revision (ICD-10) codes within 2 years from the index general health examination, health checkup questionnaires on past medical history, or corresponding measurements for each disease (cut-offs: systolic BP ≥ 140 mmHg or diastolic BP ≥ 90 mmHg for hypertension; fasting blood glucose level ≥ 126 mg/dL for diabetes; total cholesterol level ≥ 240 mg/dL for hyperlipidemia). Additionally, information on medical treatment was obtained from prescription records, and subjects who had a prescription for hypertension, diabetes, or hyperlipidemia within 2 years from the index general health examination were regarded as having the corresponding diseases.

### Outcomes

An ASCVD event was defined as the first occurrence of the composite endpoint of cardiovascular death, MI, or stroke. Cardiovascular death was identified from death records linked to the National Statistical Office using diagnostic codes; the ICD-codes I00-I99 and R96 indicated cardiovascular deaths. MI and stroke were defined based on discharge diagnosis codes from the hospitalization claims data (ICD-10 codes I21-I22 for MI and I60-I69 for stroke) [15]. In this study, it was possible to identify the occurrence of clinical outcomes until the end of 2019, allowing the development of the 10-year ASCVD risk prediction model.

### Statistical analysis

We first summarized the subjects’ characteristics by calculating means and percentages for continuous and categorical variables. We randomly split the men and women subjects in the study population into development and validation datasets at a ratio of 4:1, respectively. The prediction model was developed in the development dataset and validated in the validation dataset. A gender-specific Cox proportional-hazards regression model was employed as the base model for the development of the Korean atherosclerotic cardiovas cular disease risk prediction (K-CVD) model. To address missing data, we followed a complete-case analysis. The tentative candidate variables for the ASCVD risk prediction model included all common cardiovascular predictors that were available in the study population based on the published literature and prediction models. We also used the bootstrapping method to select statistically important predictive variables. For each bootstrapped sample, we applied the multivariable Cox model with all candidate variables and the backward variable selection approach based on Akaike information criterion values. The variables included in the prediction model had at least a 50% relative frequency in 1,000 bootstrap samples to be included. During the model-building process, clinically less-meaningful variables were sequentially excluded. We tested the interaction effect of total cholesterol and systolic BP levels with corresponding medical treatment status and included the interaction effect in the final model if there were statistically significant and clinically plausible interactions between the variables and treatment status. In the final prediction models, we examined the linearity assumption for the continuous variables using a multiple fractional polynomial model. The 10-year baseline survival probability was obtained with the Breslow estimator [16], and the 10-year risks of ASCVD were calculated based on the regression coefficients (β) and 10-year baseline survival function [S0(t)] derived from the final Cox proportional-hazards model. The performance of the derived prediction model was evaluated in terms of discrimination and calibration abilities. The discrimination ability was assessed with Harrell’s C-statistic and the time-dependent area under the curve (AUC) with 95% confidence intervals (CIs), and the calibration of the model was evaluated using the Delmer’s calibration test and calibration plots in the development and validation datasets [17]. We also calculated Brier scores as a combined measure of discrimination and calibration [18]. To demonstrate the better applicability of the K-CVD model in the Korean population, we also evaluated and compared the performance of the FRS and PCE for 10-year ASCVD risk prediction in the same cohort [5,6]. Finally, a web-based risk prediction tool for 10-year ASCVD risk was developed to facilitate the application of the K-CVD model in clinical practice.

This study followed the Transparent Reporting of a Multivariable Prediction Model for Individual Prognosis or Diagnosis (TRIPOD) guideline for the development and validation of the prediction model [19]. All statistical analyses were conducted with the R version 3.3.0 (R Foundation for Statistical Computing, Vienna, Austria). The R packages survival and APtools were used for fitting the Cox model and time-dependent AUC measures. Furthermore, the R package pec was used to calculate the Brier score. All reported p-values are 2-sided, and p-values< 0.05 were considered statistically significant.

### Ethics statement

The study protocol was approved by the Institutional Review Board of Ulsan University Hospital/Ulsan (IRB No. UUH 2018-07-008-004).

## RESULTS

### Population characteristics and outcomes

The baseline characteristics and the ASCVD event rates of the study population are shown in Table 1. In the overall population, 51.8% were men, with a mean age of 46.8± 13.5 years. Men were younger and had a higher body mass index, waist circumference, systolic BP and diastolic BP, and triglyceride levels, and lower total cholesterol and HDL-C levels than women. Men were more likely to be current smokers and less likely to receive BP-lowering and lipid-lowering medicine. During a median follow-up of 10.1 years (interquartile range, 9.5-10.5), 4,367 first ASCVD events occurred in the overall population. Cardiovascular death occurred in 1,805 (0.6%) subjects, 1,743 (0.5%) subjects had MI events, and 1,074 (0.3%) had stroke. The baseline characteristics of the study population in the development and validation dataset are shown in the Supplementary Material 1. The prevalence of risk factors was similar in the development and validation datasets.

### Development and validation of the Korean atherosclerotic cardiovascular disease risk prediction model

The gender-specific K-CVD model was developed separately from the development dataset of each gender for predicting 10-year ASCVD events in the Korean population. The included variables, coefficients, and baseline survival function for the K-CVD model are shown in Table 2. Besides the major risk factors (age, current smoking, diabetes, systolic BP, total cholesterol, HDL-C), additional variables such as urine protein for both genders and triglyceride levels for men were included in the model. Additionally, an interaction term of systolic BP and treatment status was included in models for both genders, and an interaction term of total cholesterol and treatment status was also added as a covariate in the model for women. There were no significant violations of the proportional hazards assumption in the variables of the KCVD model.

Discrimination and calibration metrics of the K-CVD model for predicting 10-year ASCVD risk are shown in Table 3. The K-CVD model had good discrimination in the development and validation datasets. For 10-year ASCVD events, the time-dependent AUC of the K-CVD model was 0.846 (95% CI, 0.828 to 0.864) in the validation dataset. The K-CVD model also showed excellent calibration ability, as evaluated by the calibration χ^2^ statistic in the development and validation datasets (calibration χ^2^ < 20 with p> 0.05 for all, indicating excellent goodness-of-fit) (Table 3).

### Comparison with the Framingham risk score model and the pooled cohort equation in the Korean population

We also evaluated the performance of the FRS and PCE for the prediction of 10-year ASCVD risk in the Korean population (Table 3). The time-dependent AUCs for FRS and PCE were similar in both the development and validation datasets (> 0.70 for all). However, these models showed poor calibration statistics in the Korean population (goodness-of-fit p< 0.001 for all). In terms of the Brier score, the K-CVD model showed the best performance with a value of 0.013, followed by values of 0.017 for PCE and 0.021 for FRS.

**Figure 2.**
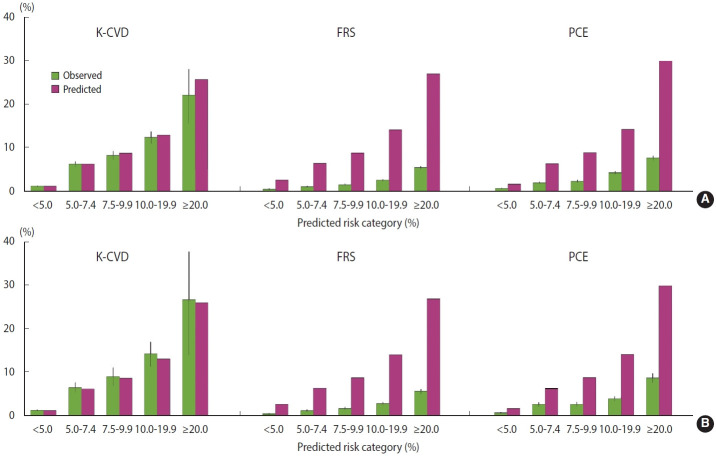
Predicted versus observed 10-year ASCVD event rates in the development and validation datasets using the K-CVD model, FRS, and PCE. Calibration plots for the predicted versus observed 10-year ASCVD event rates are shown. To assess calibration, the estimated 10-year ASCVD risk was grouped into 5 specific risk categories, and the mean predicted and observed event rates were plotted in that risk category. Predictions were made using the K-CVD model, FRS, and PCE in the development (A) and validation datasets (B). ASCVD, atherosclerotic cardiovascular disease; FRS, Framingham risk score; K-CVD, Korean atherosclerotic cardiovas cular disease risk prediction; PCE, pooled cohort equation.

The observed and predicted mean 10-year risks of ASCVD in the development and validation datasets are shown in Figure 2 and the Supplementary Material 2. In both calibration plots across 5 specific predicted risk categories and more detailed risk categories, the K-CVD model showed good agreement between the observed and predicted 10-year ASCVD rates in the development and validation datasets. Compared with the K-CVD model, both FRS and PCE overestimated the 10-year ASCVD risk in the Korean population, whereas PCE showed slightly better discrimination and calibration than FRS.

### Clinical application and real-world practice

To facilitate the clinical application of the K-CVD model, a webbased tool for 10-year ASCVD risk prediction was developed based on the coefficients and baseline survival function (https://kcvd.shinyapps.io/shiny_app_up/). The formula for calculating the 10-year ASCVD risk in the K-CVD model and an example of the risk calculation process are shown in the Supplementary Material 3. Age-specific and gender-specific 10-year ASCVD risk estimates based on varying risk factor profiles are shown in Table 4. In this simulation, subjects with optimal risk factor profiles had an estimated 10-year ASCVD risk of 0.07% to 6.31% for men and 0.02% to 5.98% for women over the age of 20 years to 80 years, whereas those with high-risk profiles had a 10-year ASCVD risk of 0.97% to 60.86% for men and 0.36% to 70.77% for women in the same age group.

## DISCUSSION

In this study, we developed and validated a 10-year ASCVD risk prediction model from a representative, contemporary, nationwide cohort of the Korean population. The newly developed risk prediction model showed excellent performance for 10-year ASCVD risk prediction in the Korean population. Our findings also confirmed that risk assessment tools such as FRS and PCE developed based on Western populations are not appropriate for the contemporary Korean population due to the overestimation of risk.

Several risk assessment tools are widely available and have been validated for predicting the 10-year ASCVD risk, including FRS, PCE, and SCORE. However, it has been questioned whether these risk assessment tools are applicable to underrepresented East Asian populations, including the Korean population [9,10]. In addition to geographic or ethnic differences, temporal trends in the epidemiology of ASCVD and risk factor profiles are other problems that have limited the generalizability of these risk prediction tools [20]. Risk prediction tools need to be periodically evaluated and validated to align with temporal trends in the prevalence of ASCVD and related risk factors in the given population. The importance of periodic recalibration can be inferred from our finding that FRS, which was derived from an earlier cohort than PCE, showed worse calibration than PCE in the contemporary Korean population. Moreover, if possible, it is preferable to develop a prediction model from a representative and contemporary population based on the local genetic characteristics and the current mix of known and unknown risk factors. However, a well-maintained and representative cohort of contemporary populations does not exist in all countries. In this study, we addressed this limitation by using the NHIS-NSC of Korea, and we believe that the approach used in our study could facilitate periodic recalibration as well as the development of prediction models in a representative contemporary population.

It has recently been suggested that widespread diabetes screening could change the cardiovascular risk profile of the population with diabetes [21]. With the increase in diabetes screening, the distribution of cardiovascular risk in subjects with diabetes is becoming more heterogeneous, and subjects with screening-detected new-onset diabetes would have different future ASCVD risk from subjects who have longstanding diabetes with microvascular complications. A similar point would hold for individuals with hypertension, which is one of the main screening targets for health screening. In this regard, an additional predictor, such as urine protein or renal function, would help to discriminate between low-subjects and high-risk subjects in the increasingly heterogeneous contemporary populations of individuals with diabetes and hypertension [22,23]. Of our candidate variables, urine protein was significantly and linearly associated with increased 10-year ASCVD risk and was included in our model. Urine protein test is a non-invasive, cost-effective method for the early detection of target organ damage, and it is commonly performed as part of routine urinalysis in clinical practice; nonetheless, it has limited specificity (i.e., elevation due to other conditions such as urinary tract infections or dehydration) and temporal variations that should be taken into account when it is used in a risk prediction model.

To account for the treatment effect of lipid-lowering and BP-lowering medication, we included corresponding treatment variables in the model in addition to traditional risk factors if relevant and significant. It is preferable to include treatment variables in the risk prediction models when models are developed from a partly treated population in which treatment status depends on the characteristics of subjects and treatments affect the outcome in the model [24]. Some modern ASCVD risk prediction models already include the use of BP-lowering treatment, but the use of lipid-lowering treatments has rarely been included in these models [25,26]. Simply ignoring treatments that have a large effect on the outcome could lead to incorrect predicted probabilities, and thus suboptimal decision-making regarding preventive interventions.

There are marked variations in risk factor and ASCVD profiles among different regions in Asia [27]. Because of their distinctive characteristics, East Asian countries have developed their own risk prediction models for their populations, and some countries have recommended the use of risk assessment tools in national guidelines for primary prevention [27]. In Korea, there have been studies to develop risk prediction models to predict overall ASCVD and its components, such as stroke and coronary artery disease [9,10,15,28]. The Korean Heart Study (KHS) developed risk prediction models for coronary heart disease and stroke [10,28]. The Korean Risk Prediction Model (KRPM) for 10-year ASCVD was also developed from the KHS cohort, which included individuals who voluntarily underwent private health examinations at 18 health promotion centers (baseline period: 1996-2001) [9]. Individuals in the KHS cohort had fewer cardiovascular risk factors than the general Korean population, and the KRPM underestimated ASCVD risks in a recent validation study based on a prospective Korean community-based cohort despite its good performance in the KHS cohort [29]. Another study developed an ASCVD risk prediction model based on general health examination data from a single center (baseline period: 2007-2011) [15]. The developed model, which was based on both traditional risk factors and biomarkers, showed a feasible model performance, but it was limited by a short follow-up duration (median, 3.1 years) and lack of population representativeness. Since there is no well-validated ASCVD risk prediction model in the general Korean population, the Korean guidelines still recommend risk stratification based on the numbers of risk factors rather than using risk prediction tools in the decision-making for drug therapy [11,12]. The strength of our study is its large sample size of the contemporary Korean population potentially eligible for cardiovascular risk assessment and the primary prevention of ASCVD. Since the target population to which the ASCVD risk prediction model is applied is temporally, geographically, and ethnically similar to the population from which the model is derived, it could be a relevant strategy to develop the ASCVD prediction model using national health examination data of the target population.

Nonetheless, the current study has several limitations. First, our study was based on a population that participated in a national health screening program. Since local subscribers or dependents could voluntarily participate in the general health examinations, unlike insured employees, the health-seeking behaviors and related cardiovascular risk factors of subjects may affect their decisionmaking on receiving general health examinations, thereby resulting in bias. In addition, between 2009 and 2010, only insured employees or self-employed householders were eligible for the general health examinations at a younger age (less than 40 years), which could also lead to bias. Second, clinical outcomes were identified from death records and hospitalization claims data. Although codes for clinical outcomes have been validated in previous studies [14,30], there is a possibility of misclassification of clinical outcomes. Third, the effects of specific types of antihypertensive or lipid-lowering medications on the risk of incident ASCVD were not considered in our model. Nevertheless, our simpler approach would be more practical in daily clinical practice. Finally, since the K-CVD model was derived from the Korean population, it is uncertain whether this model can be applied to other ethnic groups. In addition, further studies are warranted to validate its utility and determine a definite cut-off point for defining high risk, in addition to matching the intensity of preventive interventions or target goals of risk factor management to the predicted 10-year ASCVD risk. Furthermore, it should be ascertained whether incorporating additional known ASCVD risk-enhancing factors, such as women-specific conditions, chronic inflammatory conditions, or biomarkers, into the K-CVD model will improve risk stratification and guide decision-making on preventive interventions for borderline- or intermediate-risk individuals.

In this nationwide study, we developed a model for predicting the 10-year ASCVD risk in the contemporary Korean population. The K-CVD model showed excellent discrimination and calibration in Korean adults. This new population-based risk prediction tool would help to appropriately identify high-risk individuals and provide preventive interventions in the Korean population. Further studies are warranted to validate the utility of the K-CVD model in the Korean population before the model is implemented by the Korean guidelines and widely applied at the population level.

## DATA AVAILABILITY

The present study was based on the National Health Insurance Service–National Sample Cohort (NHIS-NSC) in Korea. Data are accessible to researchers after receiving permission from the National Health Insurance Sharing Service (NHISS) in Korea. Qualified, interested researchers may request access to these data from the NHISS (https://nhiss.nhis.or.kr/).

## Figures and Tables

**Figure f3-epih-45-e2023052:**
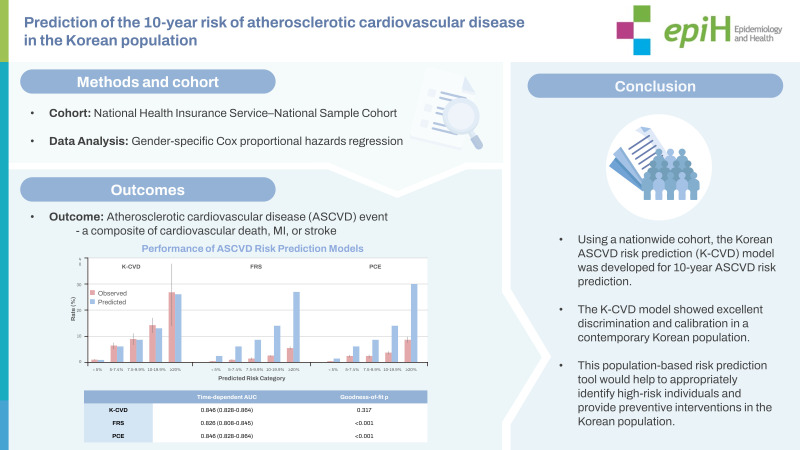


**Table 1.** Baseline characteristics and event rates of the study population according to gender

Characteristics	Overall	Men (n=168,785)	Women (n=157,149)
Percentage of total cohort	100	51.8	48.2
Age (yr)	46.8±13.5	45.8±13.2	48.0±13.7
Body mass index (kg/m^2^)	23.7±6.4	24.2 ±3.1	23.1±8.6
Waist circumference (cm)	79.9±9.1	83.6±7.8	75.9±8.8
Systolic blood pressure (mmHg)	121.9±15.0	124.5±14.1	119.1±15.5
Diastolic blood pressure (mmHg)	76.0±10.1	78.0±9.7	73.9±10.1
Hypertension	93,800 (28.8)	49,042 (29.1)	44,758 (28.5)
Hypertension on medication	53,198 (16.3)	24,606 (14.6)	28,592 (18.2)
Diabetes mellitus	39,554 (12.1)	22,115 (13.1)	17,439 (11.1)
Hyperlipidemia	77,210 (23.7)	37,384 (22.1)	39,826 (25.3)
Hyperlipidemia on medication	19,300 (5.9)	8,520 (5.0)	10,780 (6.9)
Family history of heart disease	10,017 (3.1)	4,740 (2.8)	5,277 (3.4)
Family history of stroke	18,240 (5.6)	9,08 (5.4)	9,157 (5.8)
Current smoker	81,177 (25.0)	75,634 (45.0)	5,543 (3.5)
Hemoglobin (g/dL)	13.9±1.6	15.0±1.2	12.8±1.2
Fasting glucose (mg/dL)	96.9±23.3	99.1±25.5	94.6±20.4
Total cholesterol (mg/dL)	195.8±40.6	195.4±40.4	196.2±40.7
High-density lipoprotein cholesterol (mg/dL)	56.7±30.9	53.6±29.7	59.9±31.9
Triglyceride (mg/dL)	133.7±107.4	155.1±122.4	110.7±82.5
Creatinine (mg/dL)	1.1±1.3	1.2±1.5	0.9±0.9
eGFR (mL/min/1.73 m^2^)	88.8±25.1	92.1±26.3	85.2±23.3
Urine protein			
Negative	309,905 (95.4)	160,391 (95.2)	149,514 (95.5)
Trace	7,282 (2.2)	3,816 (2.3)	3,466 (2.2)
1+	5,258 (1.6)	2,811 (1.7)	2,447 (1.6)
2+	1,890 (0.6)	1,044 (0.6)	846 (0.5)
3+	472 (0.1)	267 (0.2)	205 (0.1)
4+	98 (0.0)	61 (0.0)	37 (0.0)
Follow-up duration, median (IQR)	10.1 (9.5-10.5)	10.2 (9.5-10.5)	10.1 (9.4-10.4)
Incident clinical outcomes			
Cardiovascular death, MI, and stroke	4,367 (1.3)	2,807 (1.7)	1,560 (1.0)
Cardiovascular death	1,805 (0.6)	1,151 (0.7)	654 (0.4)
MI	1,743 (0.5)	1,344 (0.8)	399 (0.3)
Stroke	1,074 (0.3)	480 (0.3)	594 (0.4)

Values are presented as mean±standard deviation or number (%). eGFR, estimated glomerular filtration rate; IQR, interquartile range; MI, myocardial infarction.

**Table 2.** Regression coefficients and adjusted hazard ratios for 10-year ASCVD events in the K-CVD model

Variables	β coefficient	
	Men	Women
Age^1^	0.07605	0.09741
Current smoker^2^	0.63699	0.89538
Diabetes mellitus^2^	0.21904	0.29067
Systolic blood pressure^1^	0.01540	0.01353
Hypertension on medication^2^	0.25527	0.36764
Systolic blood pressure^1^ × Hypertension on medication^2^	-0.00921	-0.00634
Ln total cholesterol	0.82618	0.27455
Ln high-density lipoprotein cholesterol	-0.42561	-0.21176
Triglyceride^1^	0.00034	-
Hyperlipidemia on medication^2^	-	-5.03538
Ln total cholesterol × Hyperlipidemia on medication^2^	-	0.90851
Urine protein^1,3^	0.20974	0.28333
Baseline survival S_0_(10)	0.9995927	0.9986538

ASCVD, atherosclerotic cardiovascular disease; K-CVD, Korean atherosclerotic cardiovascular disease risk prediction; Ln, natural logarithm.

1 Centered variables; Each variable was centered at the following values (age of 40 years old, systolic blood pressure of 120 mmHg, triglyceride level of 150 mg/dL, urine protein of ≥1), respectively.

2 Binary variables where the reference value (i.e., 0) corresponds to not having that condition.

3 The urine protein level was based on dipstick urinalysis; Urine protein results of negative, trace, ≥1, ≥2, ≥3, and ≥4 were treated as corresponding ordinal variable values of 1 to 6, respectively.

**Table 3.** Comparison of the performance of the ASCVD prediction models in the development and validation datasets

Variables		Development dataset	Validation dataset
Total (n)		260,747	65,187
Actual events (n)		3,436	931
K-CVD model			
	C statistic (95% CI)	0.823 (0.813, 0.833)	0.815 (0.796, 0.834)
	Time-dependent AUC (95% CI)	0.846 (0.828, 0.864)	0.846 (0.828, 0.864)
	Calibration χ^2 1^	3.19	4.73
	p-value^1^	0.527	0.317
	Brier score	0.013	0.013
Framingham risk score			
	C statistic (95% CI)	0.804 (0.795, 0.814)	0.797 (0.778, 0.816)
	Time-dependent AUC (95% CI)	0.820 (0.801, 0.839)	0.826 (0.808, 0.845)
	Calibration χ^2 1^	92,588.23	20,912.41
	p-value^1^	<0.001	<0.001
	Brier score	0.021	0.021
Pooled cohort equation			
	C statistic (95% CI)	0.819 (0.809, 0.829)	0.813 (0.794, 0.832)
	Time-dependent AUC (95% CI)	0.841 (0.823, 0.859)	0.846 (0.828, 0.864)
	Calibration χ^2 1^	24,310.07	5,249.56
	p-value^1^	<0.001	<0.001
	Brier score	0.016	0.017

ASCVD, atherosclerotic cardiovascular disease; AUC, area under the curve; CI, confidence interval; K-CVD, Korean atherosclerotic cardiovascular disease risk prediction.

1 Calibration χ^2^ and goodness-of-fit p-values were based on 5 specific predicted risk categories.

**Table 4.** Age- and gender-specific 10-year ASCVD risk estimates based on optimal, intermediate, and high-risk factor profiles^1^

Age (yr)	Predicted 10-year ASCVD risk, %					
	Men			Women		
	Optimal RF^2^	Intermediate RF^3^	High RF^4^	Optimal RF^2^	Intermediate RF^3^	High RF^4^
20	0.07	0.29	0.97	0.02	0.08	0.36
25	0.10	0.42	1.42	0.03	0.13	0.58
30	0.15	0.62	2.07	0.05	0.21	0.94
35	0.21	0.91	3.02	0.08	0.34	1.52
40	0.31	1.32	4.38	0.13	0.55	2.47
45	0.45	1.93	6.34	0.20	0.89	3.98
50	0.66	2.81	9.14	0.33	1.45	6.40
55	0.97	4.08	13.07	0.54	2.35	10.21
60	1.41	5.91	18.53	0.87	3.79	16.08
65	2.06	8.53	25.90	1.42	6.09	24.82
70	3.00	12.22	35.50	2.30	9.73	37.14
75	4.36	17.36	47.34	3.72	15.34	53.03
80	6.31	24.34	60.86	5.98	23.75	70.77

ASCVD, atherosclerotic cardiovascular disease; RF, risk factor; HDL, high-density lipoprotein.

1 Estimated 10-year ASCVD risks for example cases with various RF profiles are shown; The definitions of the example cases are as follows.

2 Subjects with an optimal RF profile: non-smoker, absence of diabetes, systolic blood pressure of 115 mmHg, total cholesterol level of 170 mg/dL, HDL cholesterol level of 55 mg/dL, triglyceride level of 125 mg/dL, no treatment for hypertension or hyperlipidemia, negative urine protein.

3 Subjects with an intermediate RF profile: current smoker, absence of diabetes, systolic blood pressure of 150 mmHg, total cholesterol level of 200 mg/dL, HDL cholesterol level of 40 mg/dL, triglyceride level of 150 mg/dL, no treatment for hypertension or hyperlipidemia, negative urine protein.

4 Subjects with a high RF profile: current smoker, diabetes, systolic blood pressure of 160 mmHg, total cholesterol level of 250 mg/dL, HDL cholesterol level of 30 mg/dL, triglyceride level of 200 mg/dL, treatment for hypertension, no treatment for hyperlipidemia, urine protein ≥2.

## SUPPLEMENTARY MATERIALS

Supplementary Material 1. Baseline characteristics and event rates of subjects in the development and validation datasets

Supplementary Material 2 Predicted versus observed 10-year ASCVD event rates across detailed risk categories using the K-CVD model, FRS, and PCE

Supplementary Material 3 Example calculation of 10-year ASCVD risk using the K-CVD model
