# Supplementary material for: Prediction of the 10-year risk of atherosclerotic cardiovascular disease in the Korean population
Source: Epidemiol Health. 2023 May 12;45:e2023052. doi: 10.4178/epih.e2023052 (PMC10593588; doi:10.4178/epih.e2023052)
Supplement: Supplementary Material 1. — Baseline characteristics and event rates of subjects in the development and validation datasets [file epih-45-e2023052-Supplementary-1.docx]

**Supplementary material**

Sangwoo Park, et al. “Prediction of the 10-year risk of atherosclerotic cardiovascular disease in the Korean population”

**Supplemental Material 1.** Baseline characteristics and event rates of subjects in the development and validation datasets

**Supplemental Material 2.** Predicted versus observed 10-year ASCVD event rates across detailed risk categories using the K-CVD model, FRS, and PCE

**Supplemental Material 3.** Example calculation of 10-year ASCVD risk using the K-CVD model

This supplementary material was provided by the authors to give readers additional information about their work.

**Supplemental Material 1. Baseline characteristics and event rates of subjects in the development and validation datasets**

|  | Men (n=168,785) | | Women (n=157,149) | |
| --- | --- | --- | --- | --- |
|  | Development dataset (n=134,931) | Validation dataset (n=33,854) | Development dataset (n=125,816) | Validation dataset (n=31,333) |
| Age | 45.8 ± 13.2 | 45.7 ± 13.2 | 48.0 ± 13.7 | 48.0 ± 13.6 |
| Body mass index, kg/m^2^ | 24.2 ± 3.1 | 24.1 ± 3.1 | 23.1 ± 9.4 | 23.1 ± 3.3 |
| Waist circumference, cm | 83.6 ± 7.8 | 83.5 ± 7.8 | 75.9 ± 8.8 | 75.8 ± 8.8 |
| Systolic blood pressure, mmHg | 124.5 ± 14.1 | 124.5 ± 14.1 | 119.1 ± 15.5 | 119.2 ± 15.4 |
| Diastolic blood pressure, mmHg | 78.0 ± 9.7 | 78.0 ± 9.7 | 73.9 ± 10.1 | 73.9 ± 10.0 |
| Hypertension | 39,273 (29.1) | 9,769 (28.9) | 35,751 (28.4) | 9,007 (28.7) |
| Hypertension on medication | 19,709 (14.6) | 4,897 (14.5) | 22,863 (18.2) | 5,729 (18.3) |
| Diabetes mellitus | 17,334 (12.8) | 4,240 (12.5) | 13,410 (10.7) | 3,331 (10.6) |
| Hyperlipidemia | 29,953 (22.2) | 7,431 (22.0) | 31,869 (25.3) | 7,957 (25.4) |
| Hyperlipidemia on medication | 6,820 (5.1) | 1,700 (5.0) | 8,609 (6.8) | 2,171 (6.9) |
| Family history of heart disease | 3,843 (2.8) | 897 (2.6) | 4,242 (3.4) | 1,035 (3.3) |
| Family history of stroke | 7,265 (5.4) | 1,818 (5.4) | 7,339 (5.8) | 1,818 (5.8) |
| Current smoker | 60,443 (45.0) | 15,191 (45.1) | 4,445 (3.6) | 1,098 (3.5) |
| Hemoglobin, g/dL | 15.0 ± 1.2 | 14.9 ± 1.2 | 12.8 ± 1.2 | 12.8 ± 1.2 |
| Fasting glucose, mg/dL | 99.1 ± 25.6 | 99.0 ± 25.4 | 94.6 ± 20.3 | 94.8 ± 21.1 |
| Total cholesterol, mg/dL | 195.3 ± 40.5 | 195.4 ± 40.3 | 196.2 ± 41.3 | 196.0 ± 38.0 |
| High-density lipoprotein cholesterol, mg/dL | 53.6 ± 29.6 | 53.7 ± 29.9 | 59.9 ± 31.6 | 60.2 ± 32.9 |
| Triglyceride, mg/dL | 155.2 ± 124.1 | 154.4 ± 115.1 | 110.7 ± 82.1 | 110.5 ± 83.7 |
| Creatinine, mg/dL | 1.2 ± 1.5 | 1.2 ± 1.4 | 0.9 ± 0.9 | 0.9 ± 1.0 |
| eGFR, mL/min/1.73 m^2^ | 92.1 ± 26.5 | 92.1 ± 25.3 | 85.3 ± 23.4 | 85.2 ± 23.1 |
| Urine protein |  |  |  |  |
| Negative | 128,176 (95.2) | 32,215 (95.4) | 119,688 (95.5) | 29,826 (95.5) |
| Trace | 3,081 (2.3) | 735 (2.2) | 2,792 (2.2) | 674 (2.2) |
| 1+ | 2,259 (1.7) | 552 (1.6) | 1,942 (1.5) | 505 (1.6) |
| 2+ | 849 (0.6) | 195 (0.6) | 674 (0.5) | 172 (0.6) |
| 3+ | 202 (0.2) | 65 (0.2) | 168 (0.1) | 37 (0.1) |
| 4+ | 52 (0.0) | 9 (0.0) | 41 (0.0) | 6 (0.0) |
| Follow-up duration | 10.2 (9.5­–10.5) | 10.2 (9.5–10.5) | 10.1 (9.4–10.4) | 10.1 (9.4–10.4) |
| Incident clinical outcomes, n (%) |  |  |  |  |
| Cardiovascular death, MI, and Stroke | 2,209 (1.6) | 598 (1.8) | 1,227 (1.0) | 333 (1.1) |
| Cardiovascular death | 912 (0.7) | 239 (0.7) | 509 (0.4) | 145 (0.5) |
| Myocardial infarction | 1,067 (0.8) | 227 (0.8) | 311 (0.2) | 88 (0.3) |
| Stroke | 370 (0.3) | 110 (0.3) | 469 (0.4) | 125 (0.4) |

Data are mean ± standard deviation or number (%).

eGFR, estimated glomerular filtration rate; MI, myocardial infarction.
