# Supplementary material for: Prediction of the 10-year risk of atherosclerotic cardiovascular disease in the Korean population
Source: Epidemiol Health. 2023 May 12;45:e2023052. doi: 10.4178/epih.e2023052 (PMC10593588; doi:10.4178/epih.e2023052)

**Supplemental Material 2. Predicted versus observed 10-year ASCVD event rates across detailed risk categories using the K-CVD model, FRS, and PCE**

Calibration plots for predicted versus observed 10-year ASCVD event rates are shown. To assess calibration, the estimated 10-year ASCVD risk was grouped into detailed risk categories, and the mean predicted and observed event rates are plotted in that risk category. Predictions were made using the K-CVD model, FRS, and PCE in the development (A) and validation datasets (B).

ASCVD, atherosclerotic cardiovascular disease; FRS, Framingham risk score; K-CVD, Korean ASCVD risk prediction; PCE, pooled cohort equation.


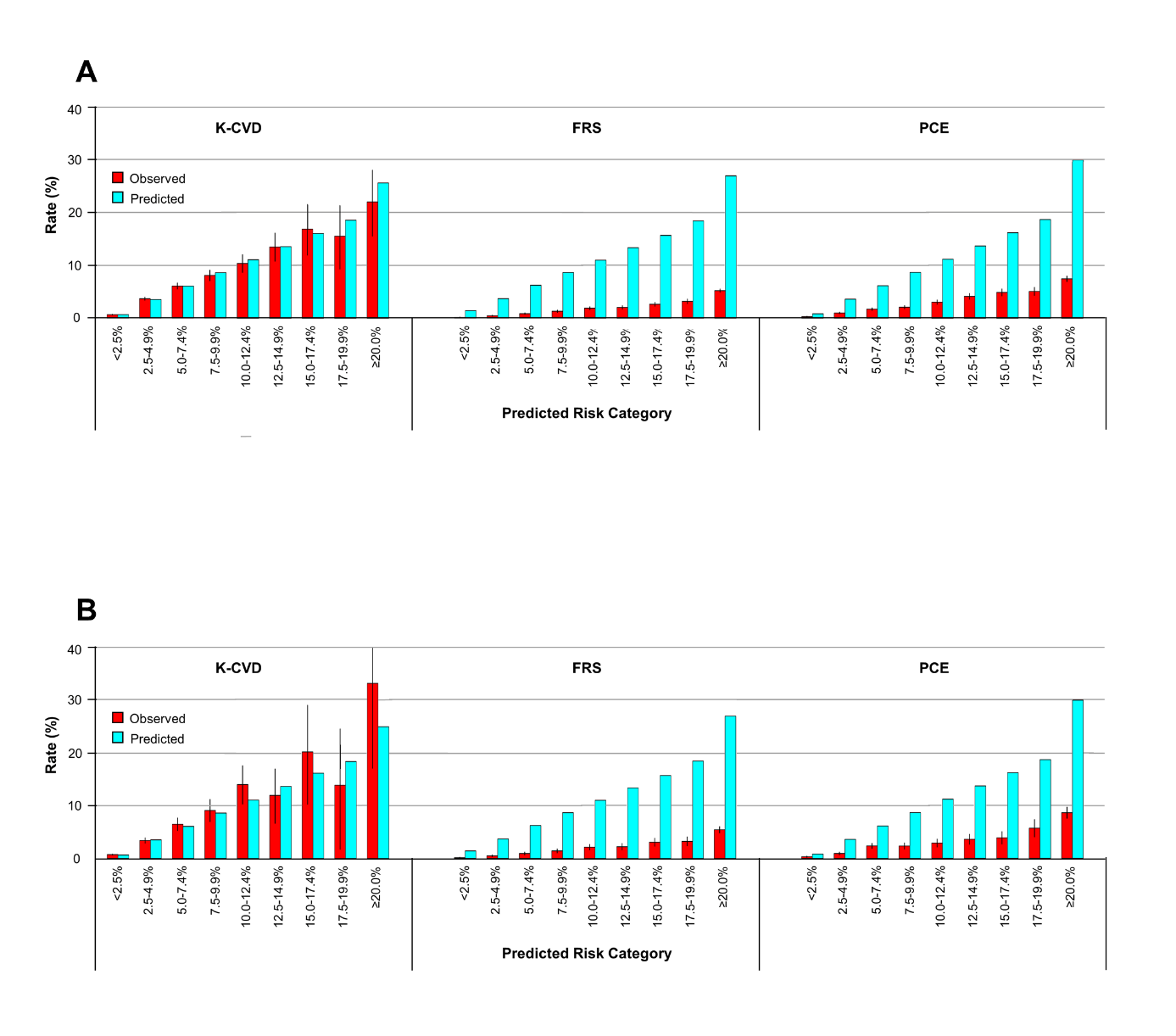

Supplement: Supplementary Material 2 — Predicted versus observed 10-year ASCVD event rates across detailed risk categories using the K-CVD model, FRS, and PCE [file epih-45-e2023052-Supplementary-2.docx]
