# Supplementary material for: Prediction of the 10-year risk of atherosclerotic cardiovascular disease in the Korean population
Source: Epidemiol Health. 2023 May 12;45:e2023052. doi: 10.4178/epih.e2023052 (PMC10593588; doi:10.4178/epih.e2023052)
Supplement: Supplementary Material 3 — Example calculation of 10-year ASCVD risk using the K-CVD model [file epih-45-e2023052-Supplementary-3.docx]

**Supplemental Material 3. Example calculation of 10-year ASCVD risk using the K-CVD model**

| **Risk calculation process** | | |
| --- | --- | --- |
| **Input data** | | |
| Subject description | A 50-year-old man/woman with a systolic blood pressure of 130 mm Hg, total cholesterol of 180 mg/dL, HDL-C of 35 mg/dL, triglyceride of 160 mg/dL, and negative result on dipstick urinalysis. The individual has diabetes. The individual is a current smoker and is not currently treated for hypertension or hyperlipidemia. | |
| **Centered variables used in calculations** | | |
| c.Age* | 50-40=10 | |
| c.SBP* | 130-120=10 | |
| c.TG* | 160-150=10 | |
| c.UrineProtein*† | 1-3=-2 | |
| **β Coefficient × variables** | | |
|  | **Man** | **Woman** |
| Age | 0.07605 × c.Age^*^=0.7605 | 0.09741 × c.Age^*^=0.9741 |
| Current smoker‡ | 0.63699 × 1=0.63699 | 0.89538 × 1=0.89538 |
| Diabetes mellitus‡ | 0.21904 × 1=0.21904 | 0.29067 × 1=0.29067 |
| Systolic blood pressure | 0.01540 × c.SBP^*^=0.1540 | 0.01353 × c.SBP^*^=0.1353 |
| Hypertension on medication‡ | 0.25527 × 0=0 | 0.36764 × 0=0 |
| Systolic blood pressure × hypertension on medication‡ | -0.00921 × c.SBP^*^ × 0=0 | -0.00634 × c.SBP^*^ × 0=0 |
| Ln total cholesterol | 0.82618 × Ln(180)=4.29032 | 0.27455 × Ln(180)=1.42573 |
| Ln HDL-C | -0.42561 × Ln(35)=-1.51319 | -0.21176 × Ln(35)=-0.75288 |
| Triglyceride | 0.00034 × c.TG^*^=0.0034 | – |
| Hyperlipidemia on medication‡ | – | -5.03538 × 0=0 |
| Ln total cholesterol × hyperlipidemia on medication‡ | – | 0.90851 × Ln(180) ×0=0 |
| Urine protein | 0.20974 × c.UrineProtein^*^=-0.41948 | 0.28333 × c.UrineProtein^*^=-0.56666 |
| **Individual sum of coefficients × variables** | 4.13158 | 2.40164 |
| **10-year risk calculation:** [1 – (S_0_(10)^exp(Sum of coefficients × variables)^)] × 100 | | |
|  | **Man** | **Woman** |
| **Baseline survival S_0_(10)** | 0.9995927 | 0.9986538 |
| **Estimated 10-year risk of ASCVD (%)** | [1 – (0.9995927^exp(4.13158)^)] × 100≈2.51% | [1 – (0.9986538^exp(2.40164)^)] × 100≈1.48% |

* Centered variables. Each variable was centered at following values (age of 40 years old, systolic blood pressure of 120 mmHg, triglyceride level of 150 mg/dL, urine protein of 1+), respectively.

† The urine protein level was based on dipstick urinalysis. Urine protein results of negative, trace, 1+, 2+, 3+, and 4+ were treated as corresponding ordinal variable values of 1 to 6, respectively.

‡Binary variables where reference value (i.e., 0) corresponds to not having that condition.

ASCVD, atherosclerotic cardiovascular disease; HDL-C, high-density lipoprotein cholesterol; K-CVD, Korean ASCVD risk prediction; Ln, natural logarithm; SBP, systolic blood pressure; TG, triglyceride.
